# Loneliness and Other Factors Associated with Physical Activity in Older Adults with Diabetes: A Cross-Sectional Study

**DOI:** 10.1177/23337214241253365

**Published:** 2024-05-10

**Authors:** Emma Cho

**Affiliations:** 1 The University of Pennsylvania School of Nursing, Philadelphia, USA

**Keywords:** loneliness, depression, diabetes, older adults, physical activity

## Abstract

The purpose of this study was to examine the association of physical activity with socioeconomic conditions, demographic factors, depression, and loneliness among older adults with diabetes in the United States. Using data from Wave 3 of the National Social Life, Health, and Aging Project (NSHAP), we found that male respondents, those with incomes greater than $100,000, and those with less depression were more likely to be physically active among older adults with diabetes. Education level and loneliness were not significant factors influencing physical activity among older adults with diabetes. This suggests that changing gender-based social norms and increasing awareness of the need for physical activity should be considered when designing physical activity interventions for older adults with diabetes and highlights the need for programs to reduce disparities in physical activity opportunities among low-income populations. It also suggests the need to further integrate programs to promote mental health, such as depression, into physical activity interventions.

Diabetes mellitus (DM) is a public health problem in the United States (U.S.), and the Centers for Disease and Prevention (CDC) estimates that approximately 11.6%, or 38.4 million, of the U.S. adult population has DM (CDC, 2022b). If left unchecked, DM can lead to serious complications such as heart disease, stroke, kidney failure, vision loss, and nerve damage (CDC, 2022a).

Physical activity (PA) is considered a cornerstone in the prevention of DM because it has been shown to significantly improve glycemic control by increasing insulin sensitivity and reducing insulin resistance (Boulé et al., 2001; Roberts et al., 2013). Regular PA also reduces the risk of DM-related complications such as heart disease and stroke by helping to maintain healthy body weight (Cox, 2017), lowering blood pressure and cholesterol levels, and improving overall physical and mental well-being (Colberg et al., 2016) and quality of life in people with DM (Soleimani Tapehsari et al., 2020).

Given the significant benefits of PA in people with DM, the American Diabetes Association (ADA) guidelines recommend that people with DM engage in at least 150 minutes of moderate-intensity activity, or 75 minutes of vigorous-intensity activity per week (Colberg et al., 2016). Despite these guidelines, only 24.1% of adults with DM met the recommended levels of PA within the past 30 days (CDC, n.d.). Understanding the factors that influence PA in older adults with DM may provide insight into developing effective interventions to promote PA and improve health outcomes in this population.

Previous studies have examined several predictors of PA, including age, gender, socioeconomic status, and psychological factors. For example, Zhao et al. (2011) conducted a study examining the association between sociodemographic factors and PA and found that older age, being female, being non-Hispanic black, and lower socioeconomic status were significantly associated with less PA engagement in adults with DM. In addition to these factors, some researchers have suggested that higher levels of depression and loneliness may also play a role in influencing PA levels in older adults with DM (Hernandez et al., 2020; Palakodeti et al., 2015).

Loneliness can be defined as the subjective feeling that can occur when there is a mismatch between the types of relationships that an individual desires and the relationships that the individual actually has (Peplau & Perlman, 1981). Approximately 38% of older adults with DM report experiencing loneliness (McCaffery et al., 2020), which may be due to the burden of managing DM and a decrease in social interactions and support (Hernandez et al., 2020; Huang et al., 2022; Kobos et al., 2020; McConatha et al., 2020). Loneliness has been associated with both psychological and physiological health outcomes in older adults with DM (Cho et al., 2024). For example, loneliness has been associated with negative perceptions of DM (Hernandez et al., 2020), depression (McCaffery et al., 2020), increased A1C levels (Huang et al., 2022), increased postprandial glucose (Avci, 2018), decreased cortisol and increased MCP-1 levels (Hackett et al., 2019), and slower walking speed and weaker hand grip strength (McCaffery et al., 2020), all of which make older adults with DM more susceptible to complications and poorer DM management.

However, despite the existing research on predictors of PA in older adults with DM, there are limitations that need to be addressed. The most common limitations of previous studies were that certain studies were not comparable to the general U.S. population due to the selection of the study population from specific regions or countries (Orzech et al., 2013; Zhao et al., 2011); one study found that the association between loneliness and physical activity was not statistically supported based on findings from a phenomenological study design (Hernandez et al., 2020). To address these limitations, the present study aims to examine the association of socioeconomic conditions, demographic factors, depression, and loneliness with PA in older adults with DM in the U.S.

## Methods

### Data and Sample

We used the publicly available data file (wave 3) of the National Social Life, Health, and Aging Project (NSHAP), which was released in 2016. In the NSHAP, participants were selected from a nationally representative, stratified, multistage probability sample of community-dwelling adults aged 57 to 85 years at the start of the first wave in 2005 to 2006. A second wave of data was then collected in 2010 to 2011 from the first wave sample and the respondents’ spouses or romantic partners living with them. In 2015 to 2016, a new, younger cohort (aged 50–67 in 2015) was added to the third wave. The sampling strategy used a clustered design with households as the primary sampling units, strategically selected from different geographic and demographic strata to ensure broad coverage of demographic and health characteristics (O’Muircheartaigh et al., 2021; Waite et al., 2021). Within each selected household, one eligible individual was randomly selected to participate. The total number of interviews in NSHAP wave 3 was 4,777. From this sample, we selected 541 respondents who were diagnosed with DM and included variables such as loneliness, depression, and PA participation. The study used publicly available, anonymized datasets and was therefore exempt from human subjects review by the authors’ institutions (O’Muircheartaigh et al., 2021).

### Measures

#### Physical Activity

The frequency of PA was measured by a single question: “On average over the last 12 months, how often have you participated in vigorous physical activity or exercise? By vigorous physical activity, we mean 30 minutes or more of things like sports, exercise classes, heavy housework, or a job that involves physical labor.” Respondents rated the frequency of their PA on a six-point Likert-type scale (0 = “never,” 1 = “less than 1 time per month,” 2 = “1–3 times per month,” 3 = “1–2 times per week,” 4 = “3–4 times per week,” 5 = “5 or more times per week”). Following the recommendations of the U.S. Department of Health and Human Services Physical Activity Guidelines for Americans (2018), respondents were categorized into three groups: low frequency (less than 1–2 times per week), moderate frequency (3–4 times per week), and high frequency (5 or more times per week).

#### Depression

Depression was measured using the 11-item shot form of the Centers for Epidemiologic Studies Depression Scale (CES-D; Kohout et al., 1993). Responses were rated on a four-point Likert scale (1 = “rarely or none of the time,” 2 = “some of the time,” 3 = “occasionally,” 4 = “most of the time”). The item referring to feelings of loneliness was excluded for this study because of its correlation with the UCLA Loneliness Scale. Two items, “I was happy” and “I enjoyed life,” were reverse scored so that higher scores indicated more severe depression. Individual item scores were summed and ranged from 10 to 40. Cronbach’s alpha for the 11-item short scale in this sample was 0.80, indicating good reliability. Convergent validity was demonstrated by correlation between CES-D-11 items and the full CES-D scale of .95 in 2,339 older adults aged 65 and older (Kohout et al., 1993).

#### Loneliness

Loneliness was measured using the 3-item form of the University of California Los Angeles (UCLA) Loneliness Scale (Russell, 1996). Responses were rated on four-point Likert scale (1 = “never/hardly ever,” 2 = “some of the time,” 3 = “often”). Individual item scores were summed such that scores ranged from 3 to 9, a higher scores indicating more frequent feelings of loneliness. Cronbach’s alpha for 3 items in this sample was 0.79, indicating good reliability. Convergent validity is demonstrated by a significant correlation of the three items from the Loneliness Scale with a single item for frequency of self-reported loneliness in the Center for Epidemiologic Studies Depression Scale (CES-D; Radloff, 1977), which is higher than the correlation between the three UCLA Loneliness Scale items and the other CES-D items, *r* = .20, *p* = .001; Hughes et al., 2004).

#### Covariates

This study included age, gender, race/ethnicity, education, and income as covariates to adjust for their potential confounding effects on the relationship between PA and health outcomes in older adults. These variables were selected based on existing literature indicating their significant influence on PA patterns. Specifically, Zhao et al. (2011) highlight the relevance of these factors in studies targeting older populations. Each covariate was operationalized as follows: age in years (a continuous variable), gender (male = 0, female = 1), education (less than high school = 1, high school or equivalent = 2, some college = 3, bachelor’s degree or above = 4), race/ethnicity (White = 1, Black = 2, Hispanic = 3, “Other” [non-Hispanic Asian American, non-Hispanic Native American/American Indian, and those endorsing non-Hispanic] = 4), marital status (currently married = 1, living with a partner = 2, separated = 3, divorced = 4, widowed = 5, never married = 6), and income (less than $24,999 = 1, $25,000–$49,999 = 2, $50,000–$99,999 = 3, and $100,000 or higher = 4).

### Data Analysis

Statistical analyses were performed using IBM SPSS Statistics (version 27; IBM Corp., 2020). Descriptive statistics were used for categorical variables. The mean and the standard deviation were used for continuous variables. The outcome variable was ordinal data categorized into low, moderate, and high frequency of PA. Kendall’s rank correlation coefficient tau-sub-b, point-biserial correlation coefficient, rank-biserial correlation coefficient, phi coefficient, and Spearman correlation coefficient were used to evaluate the relationship between PA and other variables. Ordinal logistic regression analysis was performed to identify the factors influencing the level of PA in older adults with DM.

## Results

### Descriptive Statistics

A total of 541 completed surveys were used. The mean age of respondents was 67.0 years (*SD* = 9.8), and half was male. Respondents had a variety of marital statuses. Sixty-four percent (*n* = 346) were married, others were cohabiting (4%), separated (1%), divorced (12%), widowed (12%), and never married (6.1%). Respondents were physically active at low frequency (36.6%), moderate frequency (24.8%), and high frequency (38.6%). A detailed overview of the socio-demographic composition of the study sample can be found in Table 1.

**Table 1.** Demographic Characteristics of Older Adults with Diabetes (*N* = 541).

Variables	*n* (%)	Mean (*SD*)
Age		67.0 (9.8)
Gender		
Male	267 (49.4)	
Female	274 (50.6)	
Race/ethnicity		
White	329 (60.8)	
Black	116 (21.4)	
Hispanic	72 (13.3)	
Other	24 (4.4)	
Marital status		
Married	346 (64.0)	
Living with a partner	22 (4.1)	
Separated	10 (1.8)	
Divorced	64 (11.8)	
Widowed	66 (12.2)	
Never married	33 (6.1)	
Education		
Less than high school	99 (18.3)	
High school	130 (24.0)	
Some college	188 (34.8)	
Bachelors or more	124 (22.9)	
Income		
<$24,999	133 (24.6)	
$25,000–$49,999	113 (20.9)	
$50,000–$99,999	124 (22.9)	
$100,000 or higher	63 (11.6)	
Don’t know	91 (16.8)	
Refused to answer	17 (3.1)	

*Note. n* = sample size; SD = standard deviation.

The means and standard deviations of the independent variables are presented in Table 2. Measures of loneliness and depression were self-rated. Loneliness (*M* = 4.2, *SD* = 1.5) and depression (*M* = 16.1, *SD* = 5.2) were measured using a three-point and four-point Likert scale, respectively.

**Table 2.** Means and Standard Deviations of Independent Variables.

Variables	*M* ± *SD* (range)
Depression	16.1 ± 5.23 (10–37)
Loneliness	4.2 ± 1.53 (3–9)

*Note. M* = mean; *SD* = standard deviation.

### Univariate Analysis of Physical Activity in Older Adults with Diabetes

The correlation coefficient matrix (Table 3) shows significant correlations between several variables and PA: gender (*r* = −.143, *p* < .01), education (*r* = .134, *p* < .01), income (*r* = .182, *p* < .01), loneliness (*r* = −.080, *p* < .05), depression (*r* = −.189, *p* < .01). However, age (*r* = −.048), race/ethnicity (*r* = .028), and marital status (*r* = −.068) were not significantly correlated with PA. None of the correlation coefficients suggested multicollinearity, indicating that the variables can be considered independently in further analyses.

**Table 3.** Correlation Coefficient Matrix.

Variables	Age	Gender	Race/ethnicity	Education	Income	Marital status	Loneliness	Depression	Physical activity
Age	1								
Gender	−.024	1							
Race/ethnicity	−.046	.147**	1						
Education	−.090**	−.136**	−.276**	1					
Income	−.092*	−.209**	−.277**	.372**	1				
Marital status	.078	.285**	.334**	−.182**	−.401**	1			
Loneliness	−.115**	.097*	−.024	−.055	−.179**	.225**	1		
Depression	−.125**	.149**	−.037	−.072*	−.182**	.101*	.461**	1	
Physical activity	−.048	−.143**	.028	.134**	.182**	−.068	−.080*	−.189**	1

*Note.* **p < .01, *p ≤ .05.

### Factors Affecting Physical Activity in Older Adults with Diabetes

We used ordinal logistic regression analysis to identify factors associated with the frequency of PA in older adults with DM. PA was entered as the dependent variable, and variables that were significant in the results of the univariate analysis were entered as independent variables. The ordinal logistic regression model showed a significant and good fit (*χ*^2^ (9) = 47.381, *p* = .00). Furthermore, almost 12% of the variance in the frequency of PA can be explained by the variance in the included factors (Nagelkerke *R*^2^ = .119). Table 4 provides an overview of the ordinal regression analysis. As shown, males are more likely to engage in higher levels of physical activity compared to females (beta = .47, 95% CI [−0.12, −0.04], *p* = .01). Additionally, individuals earning less than $24,999 annually are significantly less likely to participate in higher levels of physical activity compared to those with higher incomes (beta = −.85, 95% CI [−1.50, −0.20], *p* = .01). Depression also significantly influences physical activity, with higher depression scores associated with lower levels of activity (beta = −.08, 95% CI [−0.12, −0.04], *p* < .01). Education level and loneliness were not significant factors influencing PA in older adults with DM.

**Table 4.** Output Ordinal Logistic Regression Analysis of Risk Factors for Physical Activity (*n* = 541).

Variable	Estimate	*SE*	Wald	Sig. (*p*)	95% CI
Gender					
Male	0.47	0.19	6.04	0.01*	[−0.12, −0.04]
Female (ref.)					
Education					
Less than high school	1.19	0.34	0.32	0.57	[−0.47, 0.85]
High school	−0.38	0.28	1.87	0.17	[−0.94, 0.17]
Some college	0.11	0.25	0.21	0.65	[−0.37, 0.60]
Bachelors or more (ref.)					
Income					
<$24,999	−0.85	0.33	6.58	0.01*	[−1.50, −0.20]
$25,000–$49,999	−0.57	0.31	3.37	0.07	[−1.19, 0.04]
$50,000–$99,999	−0.17	0.30	0.30	0.58	[−0.76, 0.43]
$100,000 or higher (ref.)					
Depression	−0.08	0.02	12.9	0.00**	[−0.12, −0.04]
Loneliness	0.06	0.07	0.74	0.39	[−0.08, 0.20]

*Note.* Dependent variable: physical activity (low, moderate, and high frequency); SE = standard error; sig = significance.

** *p* < .01, *p ≤ .05.

## Discussion

The purpose of this study was to examine the association of PA with socioeconomic conditions, demographic factors, depression, and loneliness in older adults with DM in the U.S. First, the study found that 36.6% of older adults with DM had a low frequency of physical activity, 24.8% had a moderate frequency, and 38.6% had a high frequency. High frequency of PA is the amount of PA recommended by the ADA for older adults with DM, and this finding was higher than previously published by the CDC (24.1%; n.d.). This difference may be due to differences in the sociodemographic characteristics of the older adults with DM included in the study. For example, most studies of older adults with DM and PA include older adults aged 65 years and older (Hernandez et al., 2020; Zhao et al., 2011), whereas this study included adults aged 50 years and older. Despite the higher results in this study than previously reported, the number of older adults with DM who participate in the recommended frequency of PA is still low. This suggests that further refinement of effective interventions is needed to increase the frequency of PA needed to prevent DM complications and improve DM control in older adults with DM.

The results of this study first highlight that being male is associated with more frequent physical activity (PA) among older adults with DM. This association is consistent with previous findings, such as those of Palakodeti et al. (2015), who found that men were 1.30 times more likely than women to meet recommended levels of PA in older adults with DM. The reasons for this gender difference in PA levels remain unclear, but McKenzie et al. (2018) suggested that social norms of masculinity, which often emphasize toughness, bravery, and independence, may play an important role. Social norms refer to informally shared rules of behavior that dictate appropriate and inappropriate actions, which people follow due to societal expectations and the possibility of social penalties (Bicchieri, 2006). These masculine social norms, which are prevalent in many cultures, may encourage men to engage in PA more frequently. Therefore, the influence of social norms on PA behaviors suggests a potential avenue for intervention.

Although not limited to changing gender social norms, Van Dyck et al.’s (2011) study demonstrated that changing PA-related social norms held by family members of older adults with DM (i.e., the expectation that older adults with DM should be more physically active) led to measurable increases in PA, such as increased steps and increased use of active transportation. This suggests that interventions aimed at changing social norms may be effective in increasing PA participation in this population. Further research is therefore needed to explore how targeted changes in gender norms in our society can differentially promote PA in older men and women with DM, thereby reducing the gender gap in their PA levels. Such an approach could lead to more tailored and effective interventions that address both the physical and social factors that influence PA in older adults with DM.

Second, the study also examined the relationship between income and PA frequency. The results showed that among older adults with DM, higher income was associated with higher PA frequency, which is consistent with a meta-analysis of 19 studies (Rawal et al., 2020) and a study of 1,501 older adults in South Korea (Park et al., 2019). One possible explanation for this association is that higher incomes have greater access to resources such as gym memberships, fitness equipment, and personal trainers (King & King, 2010). In a study that partnered with community health centers to increase access to exercise for low-income diabetic adults (18 years and older) in the United States, Boyd et al. (2006) provided patients with a fee waiver and an exercise coach (pharmacist, nurse practitioner, or physician assistant). They found that exercise participation increased and clinical indicators improved as a result. While this shows that disparities experienced by older adults with DM based on income can be addressed by this intervention and suggests that it is a good model for promoting clinical measures in older adults with DM, the study focused on people with DM aged 18 years and older, which limits its generalizability to people aged 65 years and older. Therefore, further research is needed to determine whether the provision of subsidized fitness programs or community-based initiatives can help increase PA in low-income older adults with DM.

Furthermore, the association between higher levels of depression and less frequent PA is consistent with previous research (Palakodeti et al., 2015). With regard to depression, 29.2% of older adults with DM were included in the risk group for depression, so depression-related interventions should be implemented first to promote PA in older adults (Koyama et al., 2023). The relationship between depression and PA was previously reported in a study that identified predictors of PA in older adults with DM (Palakodeti et al., 2015), which supports the current findings. It was also reported that individuals with depression were 50% more likely to not meet the recommended levels of PA for older adults (150 minutes or more of moderate-intensity PA or 75 minutes or more of vigorous-intensity PA per week) compared to those without depression (Schuch et al., 2018), suggesting that higher levels of depression are associated with less frequent PA, one of the most important health-related behaviors for older adults with DM.

In addition to less frequent PA, depression in people with DM can negatively impact mental health (e.g., loneliness; Huang et al., 2022) and physical health (e.g., elevated blood glucose, elevated blood pressure), increasing the risk of complications in older adults with DM (Góis et al., 2018; Meng et al., 2012). In 2022, the national cost of DM in the United States is estimated to be $412.9 billion (ADA, 2023). Therefore, clinical strategies and interventions to prevent depression in older adults with DM should be considered to reduce the national cost of DM and the risk of DM complications and to increase PA.

In addition, education level and loneliness were not statistically significant predictors of PA in older adults with DM. This finding differs from previous research. For example, Zhao et al. (2011) compared the association between meeting PA recommendations in older adults with and without DM in the United States and found that higher levels of education were associated with a greater likelihood of meeting recommendations in older adults with DM aged 65 years, and McCaffery et al. (2020), in a study of 3,190 middle-aged and older adults with DM in the United States, reported that higher levels of loneliness were significantly associated with slower gait speed and weaker handgrip, suggesting that loneliness interferes with PA. This may be due to the age differences of the sample in the previous study and the fact that the analysis was conducted without the factors included in this study.

Another possible explanation is that depression and loneliness are highly correlated. Although the multicollinearity of these two factors was 0.5, which is less than the absolute correlation coefficient of 0.7 indicating the presence of multicollinearity (Polit & Beck, 2021), it still suggests that they may share some variance in predicting PA in older adults with DM. Therefore, future studies should examine the effect of loneliness on PA in older adults with DM while controlling for depression. Furthermore, it should not be overlooked that the synergistic effects of loneliness and depression may have a greater negative impact on the physical health of older adults with DM. Therefore, when addressing the mental health aspect of promoting active lifestyles in older adults with DM, incorporating mental health support, such as counseling or group therapy, into PA interventions might help improve overall well-being and increase exercise participation.

The strengths of this study are that it makes important contributions to academic, clinical, and professional domains. Academically, it fills a research gap by statistically analyzing the impact of loneliness on PA in older adults with DM, an area that has not been previously understood. Clinically, it highlights the importance of targeted interventions to increase PA, which may prevent DM complications and improve overall DM management. In particular, evidence that gender, income, and depression influence PA frequency provides a basis for personalized health interventions. Professionally, insights into how social norms and gender roles influence PA behaviors provide guidance for developing culturally sensitive health promotion programs that encourage older adults with DM to exercise more often. However, there are several limitations to this study. First, this study is cross-sectional, which limits its ability to establish causal relationships between individual variables and PA. Second, the physical activity and loneliness measures used in this study are a single-item and a three-item instrument, respectively, which may not fully capture the complexity and multidimensional nature of physical activity and loneliness (Hoffart et al., 2020). Therefore, future studies should consider a longitudinal design and the use of a more comprehensive physical activity and loneliness scale that can capture different aspects.

## Conclusion

In conclusion, the study found that older adults with DM had a lower frequency of recommended PA. Male gender, higher income, and lower depression were associated with a higher frequency of PA, whereas loneliness was not statistically significantly associated. This suggests the importance of changing social norms related to gender and raising awareness of the need for PA among older adults with DM. It also highlights the need for programs to reduce disparities in PA opportunities among low-income groups and suggests the need to incorporate additional mental health promotion programs such as depression into this PA intervention.
